# Developmental Trend of Subjective Well-Being of Weibo Users During COVID-19: Online Text Analysis Based on Machine Learning Method

**DOI:** 10.3389/fpsyg.2021.779594

**Published:** 2022-01-06

**Authors:** Yingying Han, Wenhao Pan, Jinjin Li, Ting Zhang, Qiang Zhang, Emily Zhang

**Affiliations:** ^1^School of Public Administration, South China University of Technology, Guangzhou, China; ^2^School of Psychology, Guizhou Normal University, Guiyang, China; ^3^College of Journalism and Communication, Guizhou Minzu University, Guiyang, China; ^4^School of Politics and Public Administration, South China Normal University, Guangzhou, China; ^5^Troy High School, Fullerton, CA, United States

**Keywords:** coronavirus disease 2019 (COVID-19), subjective well-being, latent growth mixture model (LGMM), latent growth curve model (LGCM), online text analysis

## Abstract

Currently, the coronavirus disease 2019 (COVID-19) pandemic experienced by the international community has increased the usage frequency of borderless, highly personalized social media platforms of all age groups. Analyzing and modeling texts sent through social media online can reveal the characteristics of the psychological dynamic state and living conditions of social media users during the pandemic more extensively and comprehensively. This study selects the Sina Weibo platform, which is highly popular in China and analyzes the subjective well-being (SWB) of Weibo users during the COVID-19 pandemic in combination with the machine learning classification algorithm. The study first invokes the SWB classification model to classify the SWB level of original texts released by 1,322 Weibo active users during the COVID-19 pandemic and then combines the latent growth curve model (LGCM) and the latent growth mixture model (LGMM) to investigate the developmental trend and heterogeneity characteristics of the SWB of Weibo users after the COVID-19 outbreak. The results present a downward trend and then an upward trend of the SWB of Weibo users during the pandemic as a whole. There was a significant correlation between the initial state and the development rate of the SWB after the COVID-19 outbreak (*r* = 0.36, *p* < 0.001). LGMM results show that there were two heterogeneous classes of the SWB after the COVID-19 outbreak, and the development rate of the SWB of the two classes was significantly different. The larger class (normal growth group; *n* = 1,229, 93.7%) showed a slow growth, while the smaller class (high growth group; *n* = 93, 6.3%) showed a rapid growth. Furthermore, the slope means across the two classes were significantly different (*p* < 0.001). Therefore, the individuals with a higher growth rate of SWB exhibited stronger adaptability to the changes in their living environments. These results could help to formulate effective interventions on the mental health level of the public after the public health emergency outbreak.

## Introduction

The levels of psychological stress and emotional stress, as well as the living quality of Chinese people, have been greatly altered by the coronavirus disease 2019 (COVID-19) pandemic since its initial outbreak (Cui and Kertész, 2021). The daily briefing report of the National Health Commission of China, as of May 31, 2020, reported a total of 83,017 confirmed cases in the Chinese mainland, 78,307 cured infections, and 4,634 deaths. This demonstrates a cure rate of 94.3 percent and a fatality rate of 5.6 percent (The National Health Commission of China, 2020; The State Council Information Office of China, 2020). According to an early investigation report of the World Health Organization (as of February 20, 2020), the median age of confirmed cases is 51 years (range, 2 days to 100 years old) with most cases (77.8%) aged between 30 and 69 years on the Chinese mainland. Among reported cases, 51.1% are male, 48.9% are female, 77.0% are from Hubei province, 23.0% are from other provinces on the Chinese mainland (World Health Organization, 2020). Due to the contagious nature of COVID-19 and long incubation periods experienced by patients, the Chinese government was quick to declare “war” on COVID-19 and took active steps to prevent the spread of the disease. For example, they believed large scale lockdown to be an effective way to combat the virus (Pan et al., 2020). Under the lockdown, citizens are physically isolated in their own homes and cannot proceed with offline social activities (Wang et al., 2020). Current studies found that the effective prevention and control management was related to the increased usage of social media during the COVID-19, and online social media such as Sina Weibo as the main communication media and information sources (Zhang and Yu, 2018; Li et al., 2019). This provided favorable conditions for us to explore the psychological status of Weibo users during the pandemic. Notably, in the context of the largest quasi-quarantine, this “global health crisis” will negatively affect the mental health of Chinese citizens, including their subjective well-being (SWB).

Diener et al. (1999) point out that SWB refers to individuals subjectively experiencing and evaluating their own lives; it has complex structural characteristics, which consist of life satisfaction, positive emotional experience, and negative emotional experience (Diener et al., 1999). These definitions and structures focus on personal experience in a specific social context to reflect individuals’ mental health. In this study, we focus on SWB as an overall index of psychological health. That is, those in poor mental health usually have lower SWB. Public health emergencies (e.g., COVID-19) will trigger a series of negative consequences and decrease individual SWB (i.e., psychological health). At present, some researchers have found that SWB may change as a function of the COVID-19 pandemic, but this perspective has not been well-verified in the Chinese population. Thus, during COVID-19, the changing tendency of SWB among the Chinese population deserves further investigation. Furthermore, during the COVID-19 crisis, the loss or significant reduction of SWB may increase the incidence of panic disorder, anxiety, depression, and other mental health issues (Qiu J. et al., 2020). To effectively establish psychological crisis interventions for residents, it is necessary to examine the development of SWB from the perspective of the COVID-19 pandemic.

According to previous studies, the outbreaks of COVID-19 and the related control measures can reduce SWB, but the relationship between the two is not simply linear. The research, using the questionnaire survey, found that the COVID-19 epidemic had a strong negative effect on SWB in the United Kingdom, Japan, Italy, and other countries (Banks and Xu, 2020; Papapicco, 2020; Sibley et al., 2020; Brodeur et al., 2021). The abovementioned studies did not use longitudinal investigation. However, recent longitudinal research has also found a significant negative relationship between SWB and incipient coronavirus disease 2019 outbreaks, but SWB would gradually increase as prevention and control measures were taken correspondingly. For instance, Zacher and Rudolph (2021) have shown that individuals’ SWB decreased significantly at the onset of COVID-19 outbreaks, but, after the implementation of a 1-month lockdown, the SWB of those individuals gradually increased and recovered. This result was also verified in the population groups of the United Kingdom and France (Recchi et al., 2020). In the early stages of the COVID-19 outbreak in China, there was a fierce race between the growth of the number of patients and the abundance and effective allocation of medical resources. In such cases, individual SWB will be easily influenced by the vagaries of circumstances. To investigate the existing correlation between SWB and the COVID-19 pandemic, the questionnaire method emerges as one of the most feasible strategies. However, at the time of the COVID-19 outbreak in China, it was extremely difficult to conduct a traditional paper survey in the affected areas. In addition, online surveys rely even more heavily on the cooperation of participants are difficult to meet the requirements in time and even create extra burdens for the participants. Thus, it was difficult for us to repetitively measure SWB through questionnaires at the onset of COVID-19 outbreaks. Meanwhile, The World Health Organization described the outbreak of COVID-19 as “a public health emergency” that had not been foreseen. It is impossible to measure pre-epidemic levels of SWB after the COVID-19 outbreak with questionnaires. Furthermore, there is also a notable limitation in the usage of questionnaires: responses to questionnaires can be vulnerable to some individual subjective factors, such as the emotional state, memory bias, etc. (Diener et al., 1991). Thus, we should also explore the novel method because of the numerous deficiencies of questionnaires.

Thanks to the wide usage of social media, the online text database has become a ‘‘powerful tool’’ for the real-time monitoring of changes in an emotional state, SWB, and other psychological traits of its users. Sina Weibo^1^ is one of the major Chinese online social media in the Web 3.0 era and provides users with a platform for building social networks. It is now widely used in all age groups and all 31 provincial districts in the Chinese mainland, especially the Beijing-Tianjin-Hebei region, Yangtze River Delta, and Pearl River Delta regions (Yan and Wang, 2021). More and more individuals and organizations are choosing Sina Weibo as an alternative form of real-life social networking in China. Sina Weibo owned more than 511 million monthly active users, and, among them, 45.4% were male, 54.6% were female by September 2020 (Sina Weibo Data Center, 2021). The public can express their feelings and opinions relatively freely on the Weibo platform, especially their evaluation and emotional attitudes toward their lives (Zhang and Yu, 2018). Compared with the data collected by traditional questionnaires, the online text data of social media platforms (e.g., SinaWeibo) have many obvious advantages, such as the gigantic quantity of the data, the high ecological validity, the instantaneous accessibility, a wide range of time, and lower cost of collection (Miura et al., 2015; Jones et al., 2016). Thus, online texts analysis has become an important method of studying individual psychological traits and behavioral performances.

With the progress of machine learning technology, it is possible to measure the SWB by using online text analysis from Facebook, Twitter, Sina Weibo, and other social platforms (Yang and Srinivasan, 2016). Existing studies have found that machine learning algorithm models are increasingly being used in predicting the SWB of network users (Luhmann, 2017). For example, Volkova et al. (2015) used a machine learning algorithm to analyze online text data posted by American students on Twitter and obtained the SWB level of each user (Volkova et al., 2015). Hao et al. (2014) studied the SWB level of Sina Weibo users and found that the model trained by a machine learning algorithm could predict three dimensions of the SWB more accurately, and the accuracy could reach more than 0.6 (Hao et al., 2014). By using machine learning–trained predictive models, users’ online text on Sina Weibo can be obtained to identify their psychological traits from a more ecological perspective, such as SWB. Based on web-based big data, these results of predictive models provided the entire development trend of the SWB for our research. Therefore, we can effectively examine the impact of COVID-19 and the residential lockdown on SWB from the perspective of high ecological validity.

Based on these studies, this research has two main aims: The first is to explore the trends of the SWB of Weibo users before and after the COVID-19 pandemic, and the second is to test whether there are different trajectory groups of SWB in different periods of the COVID-19. To examine the level of SWB before and after the pandemic, we obtained online texts from Sina Weibo and used machine learning algorithms to classify these online texts. Meanwhile, we used the latent growth curve model (LGCM) and the latent growth mixture model (LGMM) to explore the trends of Weibo users’ SWB and different trajectory groups of SWB during different periods of the COVID-19 pandemic. Based on existing research, we put forward the following hypotheses.

• Hypothesis 1: The subjective well-being gradually decreased during the early stages of the COVID-19 pandemic (i.e., between December 1 and December 27, 2019).
• Hypothesis 2: After the spread of COVID-19 was contained (i.e., between December 27, 2019, and April 27, 2020), the subjective well-being of Weibo users gradually increased over time, and had even exceeded the level of subjective well-being before the epidemic outbreak.
• Hypothesis 3: Since the COVID-19 pandemic outbreak, the higher the initial subjective well-being level of Weibo users, the rapid their subjective well-being level was.
• Hypothesis 4: There were multiple trajectory groups of subjective well-being across three measurement points after the COVID-19 outbreak.

## Materials and Methods

### The Selection of Active Users and Data Collection

Researchers can access massive amounts of data from the original Sina Weibo data pool quickly and cost-effectively (Wang et al., 2020). We randomly selected the online texts (Weibo ID: JncFlwj2j, Jnjwomg3z) posted by the Weibo news media, and obtained the UID of all the users that commented by a web crawler. The online texts, user UID, nickname, gender, region, brief introduction, and other information of all the commented users can be accessed through publicly available databases; the sample data can be seen in Table 1. The privacy of users was strictly protected during this process. According to Han et al. (2021), we selected active Weibo users who met the following conditions: 1. The number of online posts was more than 30; 2. The certification type was non-institutional users (such as individual users) (Han et al., 2021). Eventually, this study selected 8,748 active users and a total of 969,923 Weibo online texts. The average number of original texts by Weibo users is 244.02, the SD is 343.55, and the number of posts ranges from 31 to 3,984. The average length of each original text is 62.98 characters, the standard deviation is 124.97 characters, and the length of each original text ranges from 1 character to 4,997 characters.

**TABLE 1:**
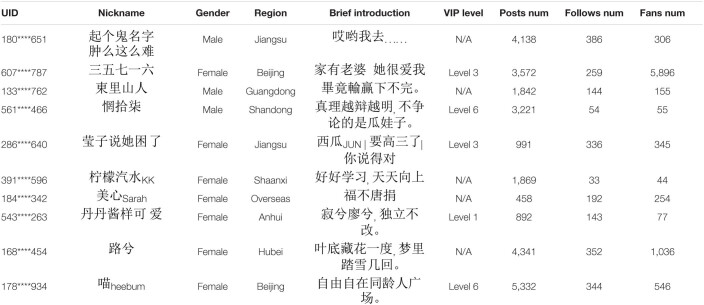
A sample of random Weibo users’ personal information.

### The Division of Pandemic Phases and Selection of Research Objects

**FIGURE 1:**
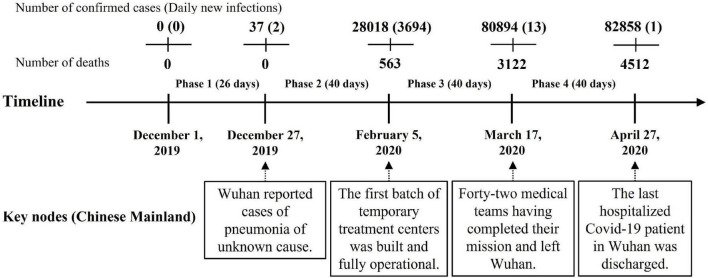
Key nodes and phases of the COVID-19 in the Chinese mainland.

This study divides the initial stage of the COVID-19 pandemic in China into four phases, with reference to *Fighting Covid-19 China in Action* (The State Council Information Office of China, 2020). They are the pre-outbreak phase (Phase 1), the outbreak phase (Phase 2), the gradual containment phase (Phase 3), and the initial victory phase (Phase 4). Figure 1 shows the timeline of these four phases and lists the key events at each time node. This timeline covers the entire process from the outbreak to the initial control of the COVID-19 pandemic in China.

Combined with the above four phases, we carried out the corresponding segmentation processing to the Weibo original texts. Meanwhile, in order to ensure the integrity and proportionality of the analytical results, we selected the Weibo users who posted during all phases of the pandemic and whose total number of posts in the four phases was at least 30. A total of 3,892 Weibo active users met the screening criteria. After eliminating content farms and undesirable personal accounts, 1,322 Weibo users (male: 509; female: 813) were counted into this study.

### Data Preprocessing and Chinese Word Segmentation

The original Weibo tweets were first screened using the Python regular expressions method (Python version 3.7.0) to pattern match texts identified as spam or invalid contents. The matching rules are shown as follows: (a) Non-original Weibo texts are removed; (b) Replace topic hashtags with a blank; (c) Remove “@username” contents; (d) Delete invalid contents, such as shared pictures, shared videos, shared articles, and shared web pages; (e) Remove all the URL links within the texts; (f) Delete all the emoticons and emojis; (g) Delete all the Arabic numbers and English characters. After preprocessing, a total of 231,210 Weibo texts remained, with an average of 174.9 Weibo texts per user.

To get the minimum particles of text analysis in the Chinese context, we used the Python word segmentation toolkit (“a Jieba Chinese word segmentation module”) to divide above Weibo original texts into words or phrases with a Jieba accurate mode. The most common and pointless stop words were removed during this process.

## Analysis Methods

### Classification and Analysis of Subjective Well-Being Levels

This study used the supervised machine learning classification model to analyze the SWB level of Weibo users based on lexical features, which comprise two main elements: the SWB lexicon and the dependable machine learning model.

We first created an SWB lexicon with high reliability and discrimination (i.e., vocabulary features). The process is described below. 1. Weibo original texts corpus construction. Weibo users were chosen randomly from the list that commented on the official posts (Weibo ID: J5zF05S2c, J5uUmsTAl, and J5u9977oo). Then, we selected the followers of the above users through snowball sampling and collected their original posts using a web crawler. After text preprocessing and Chinese word segmentation, we got a Weibo original text corpus of 11,977,831 original text corpus of 11,977,831l sampling and collected their original posts using a web crawler. After text preprocessing and Chinese word segmentation, we got a Weibo original text corpus of 11,977,831 texts. 2. Extraction of seed words. Seed words are those that can be representative of a specific domain (Li G. et al., 2020). We chose the seek words from the life satisfaction vocabulary compiled by Yang and Srinivasan (2016) and the positive and negative emotion subsets of the SC-LIWC dictionary compiled by Zhao et al. (2016). The research group also carefully screened and deleted the words that did not fit the Chinese context in the seed word list. 3. Extending new words with Word2Vec. Word2Vec is a group of related models that are used to produce word embeddings (Li G. et al., 2020). The CBOW model in Word2Vec was selected for this study to get more characteristic words of the Chinese Internet context. 4. Manual screening and construction of first edition lexicon. The first edition SWB lexicon contains 2,518 words about life satisfaction, 1,592 words about positive affect, and 2,395 words about negative affect. This SWB lexicon shows more sustainable coverage compared with the previous studies (Tausczik and Pennebaker, 2010; Mohammad and Turney, 2013), which contains not only normative vocabulary in Chinese but also social network vocabulary in the Chinese context.

Next, we induced this first edition lexicon into our machine learning classification algorithm and built a dependable machine learning model. The specific process is as follows. 1. Criteria for model comparison. We recruited Weibo users to fill out the Satisfaction with Life Scale (SWLS) and the Positive Affect and Negative Affect Scale (PANAS), and the scores of these scales were used as a criterion. 2. The supervised machine learning classification model construction. The machine learning classification model was built by comparing the algorithmic performance of four frequently and widely used machine learning algorithms such as Naive Bayes (NB), Logistic Regression (LR), Random Forest (RF), and Support Vector Machine (SVM). 3. GridSearchCV for the optimization of hyper-parameters. We found that the SVM model has higher algorithm performance after the GridSearchCV process (Best parameters: C = 10, gamma = 1.0; *Pre* score of 0.68–0.69; *Re* score of 0.67–0.69; *F*_1_-score of 0.68–0.69). This result also shows that the performance of our SVM model is better than that of other models used in related studies (Hao et al., 2014). 4. TF-IDF algorithm for lexical feature processing. After deleting the vocabulary with a feature value of 0 in the first edition SWB lexicon, we completed the final version of the SWB lexicon.

Finally, we selected the final version of SWB lexicons and the trained SVM model after GridSearchCV for classifying the SWB of Weibo original texts. The specific process is as follows. 1. The self-constructed SVM classification model and final version SWB lexicon were invoked to analyze the high-low classifications of life satisfaction, and the positive and negative effects of preprocessed Weibo original texts. And the number of high-low levels in the three dimensions of Weibo original texts was counted, respectively. 2. The high SWB was computed by summing the number of high life satisfaction original texts and high positive affect original texts, and then subtracting the number of high negative affect original texts (Conrad et al., 2010; Wang et al., 2017). After statistics, we obtained each Weibo user’s SWB level reflected by the original texts that were posted by Weibo users. 3. We counted the number of original texts with high SWB at each phase of the pandemic according to the aforementioned criteria.

### Analysis of the Latent Growth Curve Model and the Latent Growth Mixture Model

In this study, the SVM classification model based on lexical features was used to classify the SWB level of Weibo users in the four phases of the COVID-19 pandemic. Due to different attitudes toward the COVID-19 pandemic, there were individual and phase discrepancies in Weibo user’s SWB. Therefore, we adopted the LGCM and LGMM to analyze the heterogeneity characteristics of Weibo users and the developmental trend of SWB after the outbreak. The detailed analysis process was described as follows. Firstly, descriptive statistics and one-way ANOVA were conducted on the number of Weibo original texts of high-low levels in SWB to investigate the differences in the overall SWB level of Weibo users in different phases of the COVID-19 epidemic. Secondly, the unconditional LGCM was constructed to investigate the linear curve model fitting values. Meanwhile, there were significant gender differences among Weibo users (Sina Weibo Data Center, 2021). Therefore, we also included gender as a time-invariant covariate of the LGCM to analyze whether gender differences would have an impact on the SWB level of Weibo users in different phases of the COVID-19 epidemic. Finally, we constructed the LGMM to investigate the heterogeneity effect of SWB across the different Weibo users, and the developmental trend of SWB after the outbreak was investigated at the same time.

In this study, Python 3.7.0 was used to crawl and preprocess the original texts on Weibo, and it was also used to call the self-built machine learning classification model. SPSS Statistics 22.0 software was used for descriptive statistics and ANOVA analysis. The Mplus7.4 program was used for LGCM and LGMM model construction.

## Procedures

**FIGURE 2:**
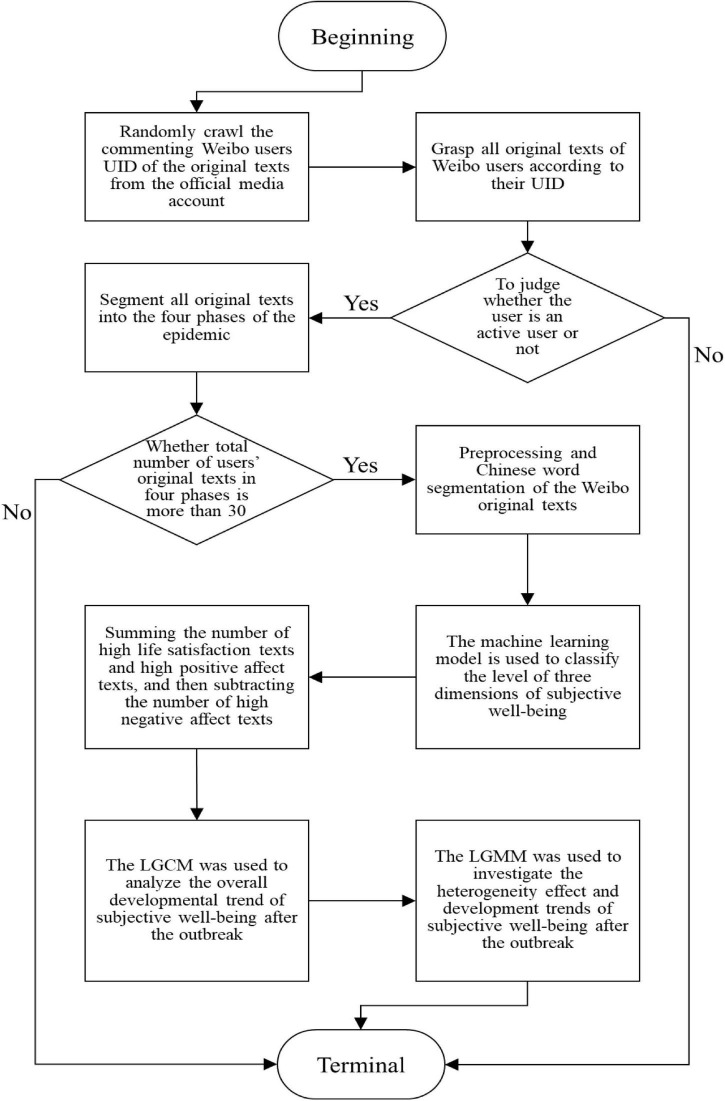
A flow chart of this study.

This study measured the dynamic characteristics of the SWB level of Weibo users during the COVID-19 pandemic. The detailed analysis process is presented in Figure 2.

## Results

The data analysis was carried out using the online texts from Sina Weibo. All online texts were divided based on the pandemic stages before analysis. SVM, descriptive statistics, LGCM, and LGMM were then used for analysis.

### Descriptive Statistics

This paper investigates the application of SVM for SWB classification. Results suggest that it is feasible to adopt SVMs for SWB classification. Descriptive statistics were used to analyze the SWB across four measurement points based on classification results. The results are shown in Table 2.

**TABLE 2:** Descriptive statistics on the number of the SWB of Weibo users in different phases of the “epidemic” (*M* ± *SD*).

Variables	Phases				*F*
	Phase 1	Phase 2	Phase 3	Phase 4	
LS	16.89 ± 20.99	13.84 ± 16.86	17.09 ± 20.92	24.95 ± 34.92	50.01***
PA	21.73 ± 29.57	17.56 ± 23.59	23.17 ± 31.69	33.40 ± 49.20	49.29***
NA	12.16 ± 16.65	11.37 ± 15.05	12.63 ± 15.53	18.04 ± 24.00	36.93***
SWB	38.73 ± 44.53	30.98 ± 35.53	39.48 ± 44.74	58.24 ± 74.31	65.83***

***p < 0.001. LS refers to life satisfaction; PA refers to positive affect; NA refers to negative affect; SWB refers to subjective well-being.

Hypothesis 1 was verified in the results. That is, the SWB of Weibo users was relatively high in the pre-outbreak phase (Phase 1), which was significantly higher than the outbreak phase (Phase 2; *p* < 0.001) and was significantly lower than the initial victory phase (Phase 4; *p* < 0.001). The analysis confirms Hypothesis 2, that the SWB of Weibo users would decrease during the outbreak phase (Phase 2), which was significantly lower than the other three phases (*Ps* < 0.001). The research results verify Hypothesis 3, demonstrating that the SWB of Weibo users increased gradually during the gradual containment phase (Phase 3) and the initial victory phase (Phase 4). Specifically, the SWB during the gradual containment phase (Phase 3) was significantly higher than the outbreak phase (Phase 2; *p* < 0.001); the SWB during the initial victory phase (Phase 4) was significantly higher than those the other three phases (*Ps* < 0.001).

### Latent Growth Curve Model Analyses

In this study, the unconditional LGCM was first used to investigate the relationship between the initial level and the development rate of Weibo users’ SWB. In the process of analysis, we firstly constructed an unconditional continuous LGCM from Phase 1 to Phase 4, but indices of fit suggested that this model did not adequately fit the data. Nevertheless, we found that SWB only decreased significantly from Phase 1 to Phase 2. Furthermore, SWB trended up during the other three phases. Based on this information, we mainly selected the data from Phase 2 to Phase 4 to build the unconditional LGCM. According to the evaluation of model fitting index, LGCM model fit indices assessed included the ratio of chi-square to degrees of freedom (*χ^2^/df*), the comparative fit index (CFI), the Tucker-Lewis index (TLI), the root mean square error of approximation (RMSEA), and the standardized root mean square residual (SRMR). The criteria of goodness-of-fit were described as follows. The *χ^2^/df* ≤ 5.00 with *p* < 0.05 (Wen et al., 2004). The CFI and TLI values > 0.90 were considered as indicators of acceptable fit (Marsh et al., 2004). The RMSEA and SRMR values < 0.08 were considered as indicators of suitable fit (Marsh et al., 1988). Inspection of the fit indices indicated a good model fit: *χ^2^/df* = 4.02, *p* < 0.001, CFI = 0.99, TLI = 0.99, RMSEA = 0.01, SRMR = 0.02 (see Table 3 for complete results). Meanwhile, the results of LGCM showed that there are individual differences in the initial level and the growth rate of Weibo users’ SWB during the epidemic. The intercept and slope means for SWB were 838.13 and 273.33 (*Ps* < 0.001), respectively. This suggested that there was a significant positive relationship between the initial level of the SWB and the growth rate of Weibo users throughout the duration of the epidemic (*r* = 0.36, *p* < 0.001). The results showed that Weibo users with a higher initial level of subjective well-being experienced a more rapid rate of increase in their subjective well-being level.

**TABLE 3:** Model fit for unconditional and covariant latent growth curve models.

Models	χ^2^/*df*	CFI	TLI	AIC	BIC	aBIC	RMSEA	SRMR
SWB (unconditional model)	4.02	0.99	0.99	40043.57	40090.25	40061.66	0.01	0.02
SWB (covariant model)	4.54	0.99	0.99	40040.90	40082.77	40061.00	0.05	0.01

*CFI refers to Comparative Fit Index; TLI refers to Tucker–Lewis Index; AIC refers to Akaike Information Criterion; BIC refers to Bayesian Information Criterion; aBIC refers to Sample-Size Adjusted Bayesian Information Criterion; RMSEA refers to Root Mean Square Error of Approximation; SRMR refers to Standardized Root Mean Square Residual.*

Since there were significant gender differences among Weibo users, we further included gender as a covariant, examining gender differences in the developmental trajectory of Weibo users’ SWB. The LGCM included gender variables as time-invariant covariates, and the results showed an excellent model fit:χ^2^/*df* = 4.54, *p* < 0.001, CFI = 0.99, TLI = 0.99, RMSEA = 0.05, SRMR = 0.01 (see Table 3 for complete results). The results present the regression coefficients of the intercept factor and slope factor means for gender to be −0.03 (*P* = 0.28) and 0.01 (*P* = 0.85), respectively. This demonstrates that the slope and intercept were not significantly different; thus, there is no significant difference in the SWB of Weibo users based on gender. The intercept factor remained significantly positively correlated with the slope factor after controlling for the gender covariate (*r* = 0.42, *p* < 0.001), which indicated that the higher the initial level of Weibo users’ SWB of different genders, the faster growth rate of their SWB level.

### Latent Growth Mixture Model Analyses

#### Potential Class Analysis of Subjective Well-Being After the Outbreak of the Pandemic

We examined a series of LGMM analyses including an increasing number of latent classes. Fit indices of each class are shown in Table 4. Model fit statistics were calculated to identify the optimal number of trajectory classes (Stern and Hertel, 2020), including AIC, BIC, aBIC, Entropy, LMR, and BLRT. Results of the LMR and BLRT point out a two-class solution. Additionally, a two-class solution exhibited relatively low AIC, BIC, aBIC, and relatively high Entropy value, which suggests a better fitting model. Furthermore, models with more than 3 classes included very small classes (i.e., <3% of the sample). Thus, the two-class model was selected as the best fitting model of SWB. In a LGMM, each individual receives an estimated probability for belonging in each of the classes (average posterior probabilities). In this study, the average posterior probability for individuals better matches two latent classes, as shown in Table 5. The average posterior probabilities for individuals belonging to the respective class were >95% in all the classes.

**TABLE 4:** Fit indexes for determining optimal number of latent classes.

Class	K	Log(L)	AIC	BIC	aBIC	Entropy	LMR(*P*)	BLRT(*P*)	Conditional probabilities
C1	9	-20010.134	40038.27	40084.95	40056.36	N/A	N/A	N/A	N/A
**C2**	**12**	-**19614.072**	**39252.15**	**39314.39**	**39276.27**	**0.98**	**0.03**	**<0.001**	**0.937/0.063**
C3	15	-19329.220	38688.44	38766.24	38718.60	0.99	0.58	<0.001	0.059/0.010/0.931
C4	18	-19160.985	38357.97	38451.34	38394.16	0.98	0.24	<0.001	0.044/0.911/0.042/0.004
C5	21	-19064.019	38170.04	38278.96	38212.26	0.99	0.22	<0.001	0.005/0.906/0.032/0.053/0.003

*K refers to a number of freely estimated parameters; Log (L) refers to Loglikelihood; LMR refers to Lo–Mendell–Rubin Likelihood Ratio Test; BLRT refers to Bootstrap Likelihood Ratio Test. The bold values represent those of the best fitting model.*

**TABLE 5:** Average ascription probability (column) of participants in each latent class (row).

	C1 (%)	C2 (%)
C1	95.2	0.3
C2	4.8	99.7

#### Developing Trends of Different Classes of the Subjective Well-Being After the Outbreak of the “Epidemic”

There are two heterogeneous classes of the trends of Weibo users’ SWB during the outbreak of the epidemic. Based on this, this study further examined the dynamic change of two heterogeneous classes. With the LGMM, two latent variables were obtained for each class, namely the individual level parameter (i.e., intercept) and the growth rate parameter (i.e., slope). The first parameter (the intercept factor) describes the initial level of SWB (intercept mean) and individual differences in the initial level (intercept variance). The second parameter (the slope factor) describes the rate of change (slope mean) and individual differences in growth patterns (slope variance).

From Table 6, the intercept mean did not significantly differ between the two classes during the outbreak of the epidemic (*Ps* > 0.05). But an examination of the two classes extracted revealed one small class (C1, *n* = 93, 6.3%), which showed a higher initial level of SWB. Additionally, the results obtained in this study reveal that the slope means across the latent two classes were significantly different (*p* < 0.001), suggesting that the average growth rate of the SWB was higher in the C1 class than the C2 class. The correlation between the intercept factor and the slope factor was not significant (*Estimate* = 139.02, *p* = 0.03). Our results also showed that there was a significant positive correlation between the initial level and the growth rate of Weibo users’ SWB in both classes.

**TABLE 6:** Intercept and slope mean values of each latent class.

	Intercept			Slope		
	Estimate	S.E.	*P*	Estimate	S.E.	*P*
C1	14.49	8.50	0.09	2.88	0.81	<0.001
C2	2.60	1.44	0.07	0.57	0.14	<0.001

**FIGURE 3:**
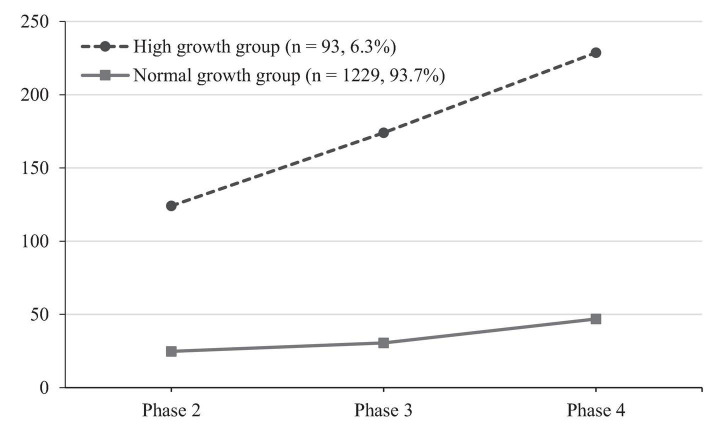
Two latent classes of a developmental trend of subjective well-being (SWB) after the COVID-19 outbreak.

Based on the developmental shifts in the specific features of Weibo users’ SWB in two classes, the C1 class is named the “High growth group,” representing 6.3% of the total sample. The SWB of C1 class showed a faster upward trend during the outbreak. C2 class is named the “Normal growth group,” accounting for 93.7% of the total samples, and their SWB showed a slower increase during the COVID-19 pandemic (see Figure 3 for details).

## Discussion

This study carried out a longitudinal study on SWB with the machine learning method to investigate the trends and multiple trajectory groups of the SWB during the pandemic in China.

The results of the one-factor ANOVA revealed that SWB in the early period of the COVID-19 outbreak was significantly lower than SWB before the outbreak. This result is consistent with the research results of Germany, the United Kingdom, and France, that is, the SWB of individuals was significantly decreased at the initial phase of the lockdown (Recchi et al., 2020; Valdez et al., 2020; Zacher and Rudolph, 2021) when China had no effective treatment and vaccine in the early period of the COVID-19 outbreak. Therefore, concerns related to the morbidity and the mortality rate of the disease may have led to the decrease of SWB. In addition, the “self-isolation” and “social distance” brought by the lockdown may have intensified and exacerbated the diminishing state of well-being (Terrizzi et al., 2013; Banerjee, 2020; Li S. et al., 2020; Valdez et al., 2020). On the whole, the strict regulations of community lockdowns and the limitations on offline social activities did have some detrimental impacts on SWB in the early stage of the COVID-19 pandemic. Follow-up studies further discovered that, after the implementation of lockdown drills, the SWB of individuals would gradually increase and eventually rebound to the level before the outbreak with effective prevention and control measures (Recchi et al., 2020; Wang et al., 2021; Zacher and Rudolph, 2021). A large-scale, longitudinal investigation, which was conducted among 18 U.S. cities, also found that there were three typical stages of psychological transformation, namely the “warning phase,” “isolation phase,” and “normalization phase,” and most psychological transformations were stronger when the threat of COVID-19 increased (Ashokkumar and Pennebaker, 2021). This study might further confirm the above research. In addition, we found that the SWB increased significantly after the implementation of the lockdown for a while, and even exceeded the level of SWB prior to the pandemic. The causes of this phenomenon might be including the following.

First, the increase of SWB was closely related to the prevention and control measures of the Chinese government. In the early phase of the outbreak, it was necessary to cut off “human-to-human” transmission through containment measures. When the pandemic first started in early 2020, the Chinese government adopted the policies of mandatory quarantine and large-scale lockdowns, and strictly prohibited all activities that were detrimental to their prevention and control measures. At the same time, all primary-level organizations in China were quick to adopt these government-issued prevention and control measures to appeal to the government. As a result, it took only 1 month to develop a nationwide prevention campaign in China. From the later period of the epidemic in China, it is evident that the prevention and control measures of the Chinese government have achieved satisfactory results (Anderson et al., 2020; Qiu Y. et al., 2020; Wang et al., 2021). Second, collectivism might be one of the important reasons for China’s success in the prevention and control of the pandemic and an increase in residential SWB. There is general agreement that China is a typical paradigm of a collectivistic society (Alkhadher et al., 2020; Ren et al., 2021). In collectivist values, individuals tend to regard groups and communities as the core of social units, which emphasize social relationships and self-identity in a group (Hofstede, 1980). And individuals in collectivist societies tend to use the power of the collective for reducing the perceived stress when facing stressful events (Ren et al., 2021). Therefore, it is not difficult to understand why China was able to receive many forms of internal assistance during COVID-19–this country believes that “when disaster struck, help came from all sides.” For instance, all the other provinces in China raced against the clock to assist Hubei province after the COVID-19 outbreak. Current studies suggest a positive and significant relationship between individual well-being and collectivism (Rathi and Lee, 2021). Jiang et al. (2020) pointed out that collectivistic countries, such as China and South Korea, are confident to block the spread of the COVID-19 epidemic. However, individuals in individualistic countries, such as the United Kingdom and the United States, had higher independence and autonomy (Yin et al., 2019). In these countries, the strict prevention and control measures (e.g., lockdown) may fail, and the government might face enormous pressure of public opinion and a grim challenge of COVID-19 prevention and control (Jiang et al., 2020).

The above results were only based on simple differential analysis, and we cannot know the initial level and the growth rate of the SWB through ANOVAs. Therefore, we used the LGCM model to further analyze Weibo users’ SWB during the COVID-19 pandemic. Several findings of this study revealed that the higher the initial level of Weibo users’ SWB, the faster the increase of their SWB. The theory of character strengths indicates that those with high SWB are good at making use of their character strengths to give themselves more profound experiences of happiness (Koch et al., 2020). Specifically, typical character strengths of individuals include hope (e.g., optimism), love (e.g., giving and receiving love and care), and zest (e.g., vitality and enthusiasm). Meanwhile, the tendency of optimism was one of the most important traits of high-SWB groups (Carver et al., 2010). Optimists tend to expect events to turn out well, and they will experience more positive emotions (Carver and Hoyle, 2009). For instance, when encountering difficulties, optimists would likely possess the mindset of “The conditions are better than expected” rather than “The conditions are not as good as expected” (Lee and Mason, 2013). Meanwhile, they are used to adopting a more proactive approach and trying their best to solve problems (Carver and Hoyle, 2009). The resource protection model also emphasizes that the ability to use the character strengths can elevate individual SWB even under situations of high stress like the COVID-19 pandemic, that is, the SWB level of individuals with a proficient ability to use their character strengths (individuals with a high initial of SWB) will be significantly higher than those incapable of using these advantages (individuals with a low initial level of SWB). In other words, it is precise because the group with high initial SWB makes effective use of their character strengths (optimism) that their SWB increases rapidly. And it could explain why those with a higher initial level of SWB will have a higher growth for SWB during the epidemic. Genç and Arslan (2021) also suggested that the optimistic tendency can relieve adverse effects of the COVID-19 on the SWB. During the COVID-19 outbreak, the optimistic tendency can enhance SWB by promoting positive emotions and using positive coping strategies (Joshanloo et al., 2017). In addition, a significant gender difference was found in the selected Weibo users, so we constructed the LGCM model and used gender as a covariant. However, the initial level and the growth rate of the SWB had no significant difference between male and female Weibo users, which was consistent with previous studies (Upasna, 2010). We found that the adaptability to negative events could explain why there was no gender difference in the SWB. A possible explanation may be that the adaptability and the initial SWB between men and women are similar. Life events do not affect their adaptability but may have a temporary adverse impact on their SWB (Luhmann et al., 2012; Wang et al., 2021). Therefore, even if they underwent COVID-19, their SWB will recover to the previous level over time under the influence of adaptability. On the whole, the similar adaptability and the similar initial SWB is the reason why there is no gender difference in the level of SWB.

Latent growth mixture model was an effective technique for identifying multiple trajectory groups of the SWB. Our multiple group analysis indicated that there were two clear and distinct patterns of SWB of Weibo users. The larger group (*n* = 1,229, 93.7%) showed a slow growth in the SWB, which can be named the normal growth group. The other group (*n* = 93, 6.3%) had more rapid growth in their SWB and can be called the high growth group. By reviewing previous studies, we speculated that the different growth rates of the SWB between two groups might be caused by the following reasons. First, the high growth group may have a stronger collectivism tendency compared with the normal growth group, and collectivism was found to predict SWB. Although China is a typical country with a collectivism culture, studies have shown that collectivism also has individual differences (Lu et al., 2021). Kim et al. (2016) believe that social members with a high collectivism tendency have a greater sense of belonging and closer social relationships, creating a buffer zone against the pandemic, and their SWB can, in turn, be improved (Kim et al., 2016; Ahuja et al., 2020). In other words, in collectivistic societies like China where joint families and closer social relationships are thought to be important social resources, these resources may have contributed to the maintaining of an individual’s SWB during the COVID-19 pandemic. This effect is particularly noticeable in the high growth group. Second, the high growth group might have stronger ethnic and national loyalties compared with the normal growth group. Although the prevention and control measures have been proved effective (Anderson et al., 2020; Qiu Y. et al., 2020), some countries lagged behind in executing them. For example, leaders of the United States, Brazil, and Mexico were all reluctant to adopt quarantine and lockdown measures during the early stages of the epidemic, even in the face of rapidly growing numbers of new COVID-19 cases in their countries (Fukuyama, 2020). This inaction directly caused the continuous spread of the global epidemic, in sharp contrast to China, which has achieved initial success in controlling the spread of the epidemic. In addition, Chinese social media released a lot of positive reports on the prevention and control of the epidemic, which increased the confidence and positive cognition of the Chinese population. Therefore, SWB has different levels of increase in different populations. Accounting for individual differences, the growth rate of SWB is also different among individuals. In addition, the SWB is positively associated with patriotism (Kimhi et al., 2020), especially when the pandemic is in a positively recovering trend. Thus, the high growth group might be a small number of people who have strong ethnic and national loyalties. They are fanatical Chinese patriots and have a strong national pride, so their SWB increased rapidly after COVID-19 was contained, and the positive media information appeared (Yang et al., 2020; Chu et al., 2021).

## Limitations and Future Directions

Although we collected a considerable amount of text for examining the SWB here, there are still several limitations to this study. First, despite the stricter preprocessing program adopted in this study, there remains some irrelevant information in Weibo user’s original texts. Future research could further optimize the cleaning method of original texts and retain the texts with higher analytical value. Second, the supervised machine learning model was adopted to classify original texts in this study. But this kind of model requires huge numbers of labeled data as references, which may be affected by individual differences in inner experiences and contextual comprehension. Future studies can explore unsupervised machine learning algorithms in text classification such as the convolutional neural network. Third, this study mainly explored the longitudinal changes of SWB after the COVID-19 outbreak, but it is worthy to explore and investigate whether there was a causal relationship between other variables and the trajectories of SWB. Therefore, future studies could focus on the factors affecting the trajectories of SWB after the COVID-19 outbreak. Finally, the results of our research face the challenge of ecological validity. Even though our research dataset was collected from one of the most popular social media platforms in China, there were still some problems making the results hard to generalize to larger populations and more linguistically diverse environments. For example, our research was mainly applicable to groups with eastern cultural backgrounds, which might be different from the results in western cultural backgrounds. Therefore, future studies may be performed for the dataset from multiple online social platforms (i.e., Twitter, Facebook) to obtain more comprehensive results, to enhance the application value and promotion value of research results.

In spite of the above limitations, the present research has also made some contributions to existing studies in the field of SWB and also has implications for research and practice. Firstly, different from those previous studies with psychological questionnaires, the present study concentrated on the network heterogeneity and the trajectories of SWB on social media during the public health emergency. To the best of our knowledge, this is the first study to focus on the heterogeneous growth trend of the SWB on social media during the COVID-19. Second, the present study combined machine learning with LGMM to analyze the heterogeneity and trajectories of SWB on social media, which could be useful for improving prediction accuracy. This study found that there were two potential classes of the trajectories of SWB and that the individuals in the high growth group exhibited stronger adaptability to the changes in their living environments than those in the normal growth group, which corresponds and directly relates to the faster growth rate, or recovery rate, of their SWB. These findings could help to formulate effective interventions on the mental health level of the public after the COVID-19 outbreak.

## Data Availability Statement

The raw data supporting the conclusions of this article will be made available by the authors, without undue reservation.

## Ethics Statement

Ethical review and approval was not required for the study on human participants in accordance with the local legislation and institutional requirements. Written informed consent for participation was not required for this study in accordance with the national legislation and the institutional requirements.

## Author Contributions

YH was involved in the conception and research design. WP was involved in the research design, collection, and assembly of the data. JL was involved in the writing of the manuscript. TZ was involved in the data analysis and interpretation. QZ and EZ was involved in the reviewing of the manuscript, and comments response in the process of revising the manuscript. All authors contributed to the article and approved the submitted version.

## Conflict of Interest

The authors declare that the research was conducted in the absence of any commercial or financial relationships that could be construed as a potential conflict of interest.

## Publisher’s Note

All claims expressed in this article are solely those of the authors and do not necessarily represent those of their affiliated organizations, or those of the publisher, the editors and the reviewers. Any product that may be evaluated in this article, or claim that may be made by its manufacturer, is not guaranteed or endorsed by the publisher.

## Footnotes

1
https://weibo.com
